# A Rasch Analysis of the Charcot-Marie-Tooth Neuropathy Score (CMTNS) in a Cohort of Charcot-Marie-Tooth Type 1A Patients

**DOI:** 10.1371/journal.pone.0169878

**Published:** 2017-01-17

**Authors:** Wenjia Wang, Mickaël Guedj, Viviane Bertrand, Julie Foucquier, Elisabeth Jouve, Daniel Commenges, Cécile Proust-Lima, Niall P. Murphy, Olivier Blin, Laurent Magy, Daniel Cohen, Shahram Attarian

**Affiliations:** 1 Inserm U1219, Université de Bordeaux, Bordeaux, Aquitaine, France; 2 Pharnext, Issy-Les-Moulineaux, France; 3 CIC-CPCET, Service de Pharmacologie Clinique et Pharmacovigilance, AP-HM, Aix Marseille Université, Marseille, France; 4 Centre de Référence Neuropathies Périphérique Rares, CHU de Limoges - Hôpital Dupuytren, Limoges, France; 5 Aix Marseille Université, INSERM, GMGF, Marseille, France; Taipei Veterans General Hospital, TAIWAN

## Abstract

The Charcot-Marie-Tooth Neuropathy Score (CMTNS) was developed as a main efficacy endpoint for application in clinical trials of Charcot-Marie-Tooth disease type 1A (CMT1A). However, the sensitivity of the CMTNS for measuring disease severity and progression in CMT1A patients has been questioned. Here, we applied a Rasch analysis in a French cohort of patients to evaluate the psychometrical properties of the CMTNS. Overall, our analysis supports the validity of the CMTNS for application to CMT1A patients though with some limitations such as certain items of the CMTNS being more suitable for moderate to severe forms of the disease, and some items being disordered. We suggest that additional items and/or categories be considered to better assess mild-to-moderate patients.

## Background

Charcot-Marie-Tooth (CMT) disease is the most common inherited disorder of the peripheral nervous system [1,2]. CMT type 1A (CMT1A), caused by a duplication of the myelin protein encoding gene *PMP22* [3,4], accounts for 50% of patients with CMT [1,2,5]. A typical feature of CMT1A is weakness of the foot and lower leg muscles, which may lead to foot drop and a high-stepped gait with frequent tripping or falls. Currently, there are no approved treatments for CMT1A disease though there have been considerable interest in the potential of ascorbic acid (AA) as a therapy leading to six clinical trials investigating the efficacy of AA on CMT1A [6–11]. Unfortunately, no beneficial clinical effects of AA were identified in any of these trials, as confirmed by two meta-analyses [12,13]. Recently, a clinical trial of PXT3003 (a fixed combination of baclofen, naltrexone and sorbitol) showed preliminary evidence of efficacy in an exploratory phase 2 study [14], which was also confirmed by a meta-analysis [12]. A conclusion shared by all of these studies is that selecting a clinically meaningful efficacy endpoint for CMT1A trials is challenging. Among the reasons for this are questions surrounding the relevance of efficacy endpoints, which remains an active topic of discussion. With regard to this, the Charcot-Marie-Tooth Neuropathy Score (CMTNS) was first proposed and validated by Shy *et al*. to provide a reliable measure of impairment in CMT [15]. The CMTNS is composed of 9 items evaluating different functions related to the disease: 5 of impairment (‘*Sensory Symptoms*’, ‘*Pin Sensibility*’, ‘*Vibration*’, ‘*Strength Arms*’ and ‘*Strength Legs*’), 2 of activity limitations (‘*Motor Symptoms Arms*’ and ‘*Motor Symptoms Legs*’) and 2 electrophysiological measures (‘*Ulnar CMAP*’ and ‘*Ulnar SNAP*’). Each item is scored from 0 to 4 and the total sum of the item scores provides a global measure of disease severity, with higher scores indicating worsening function [15].

The CMTNS has been used as the primary or main endpoint in most completed clinical trials for CMT1A to date. However, the ability of the CMTNS to measure responses to treatment has not been demonstrated and among all the studies published, meta-analysis reveals significant improvements on the CMTNS only under PXT3003 versus placebo [12]. With this in mind, the sensitivity of the CMTNS to change and its psychometric properties are still debated. In particular, it has been suggested that some components of the CMTNS are too insensitive, mainly because of floor and ceiling effects [16]. Therefore, a modified version of the scale (CMTNS-v2) has been proposed by Murphy *et al*. [17] in an attempt to reduce the aforementioned effects and to standardize patient assessment. This version has also been questioned recently and a ‘weighted’ alternative has been suggested as a potential improvement [18]. Finally, a modified CMTNS (called CMTNS-Mod) has also been proposed by adding three functional measures (9-hole peg test, foot dorsiflexion and walk test) while removing four of the initial items (‘*Ulnar SNAP*’, ‘*Pin Sensibility*’, ‘*Vibration*’ and ‘*Strength of Arms*’) [19]. However, none of these modified versions have been evaluated in natural history or therapeutic trials. As the CMTNS is the only CMT specific outcome measure available and has been widely applied, it is important to review its properties and find directions in which it could be improved. Firstly, it is important to demonstrate the sensitivity of CMTNS scores with disease progression. With regard to this, the CMTNS showed modest changes over time in longitudinal studies. Shy *et al*. reported a mean increase of 0.69 points per year for natural progression [20]. In parallel, clinical trials showed that CMT1A under placebo deteriorates even more slowly with a mean increase of 0.16 points per year [12].

To be a valid indicator of disease severity, the CMTNS should also comply with requirements of modern measurement theory such as unidimensionality (which implies that the scale measures only one construct, which allows the items to be summed together to form a scale with only one dimension), internal construct validity and reliability [21]. One well-accepted way to provide such evidence is to perform a Rasch model analysis [22], which has been widely employed in clinical scale construction and validation [21,23,24]. The Rasch Model assesses a latent trait, such as disease severity, by the responses of patients to a set of items [25]. It provides a range of diagnostic information that can be used to determine how well each item contributes to the measurement of the latent trait and doing so, it helps in assessing the validity of the scale and its possible axes for improvement.

Here, we performed a Rasch analysis of the CMTNS in a cohort of 277 mild-to-severe CMT1A patients from merging of two French clinical trials [9,14] and one non-investigational study.

## Methods

### Participants and setting

CMT1A patients involved in this study initially participated in the French phase 2 clinical trial of ascorbic acid led by Micallef *et al*. [9] and/or in the phase 2 clinical trial of PXT3003 led by Attarian *et al*. [14] and/or in a subsequent non-investigational clinical study (BMK-CMT) sponsored by Pharnext. The ethics committee "Comité de Protection des Personnes Sud-Méditerranée I" has approved this study and the RCB ID is 2010-023097-40. Participants provide their written informed consent to participate in the study. Patients were included from six hospital sites in France: Marseille, Lille, Limoges, Lyon, Nantes and Paris. A total of 277 patients completed the CMTNS scoring in at least one of the 3 aforementioned clinical studies. Among them, there were 110 men and 167 women, with ages ranging from 18 to 69 and an average age of 45. Patient CMTNS scores ranged from 2 to 31 and were classified into mild (CMTNS ≤ 10, 47 patients), moderate (11 ≤ CMTNS ≤ 20, 201 patients) or severe (CMTNS ≥ 21, 29 patients).

### The Rasch model

The Rasch model is a mathematical framework initially proposed to analyze rating scales and evaluates a latent variable not measurable directly from a set of categorical items (eg, disability, cognition or quality of life). In this model, the raw score of each item is transferred into interval scaling by a logistic function where data are found to meet the model assumptions. Both the person’s ability and the item difficulty, also referred to as person and item parameters, are defined on the same dimension. If a person’s ability is known, it is possible to predict how that person is likely to perform on a given item. Specifically, the probability of a response is modeled as a logistic function of the difference between the person and the item parameters. The Rasch model was initially developed for dichotomous items, and then extended to polytomous items in which successive integer scores represent categories of increasing level of disability such as the CMTNS.

### Rasch model analysis

The Rasch model analysis provides an integrated framework for evaluating if a sum score (such as the CMTNS) satisfies a set of requirements listed hereafter. They first include the three assumptions of the Rasch Model, that of local independence, unidimensionality and invariance, briefly explained as follows:

Local independence means that, conditionally on the latent person ability, the response of a particular individual to an item depends neither on the responses to other items nor on the responses given by other people to the same item. This is examined by the residual correlations between items, which should be no more than 0.3 for each pair of items [26].

#### Unidimensionality

The Rasch model assumes that the response to each item depends on a unique latent trait. It can be assessed by creating two subsets of items using a Principal Component Analysis (PCA) of the item residuals, with those loading negatively forming one set, and those loading positively forming the second set. Each person parameter estimated from one set of items is then compared to those derived from the other set of items using a *t*-test. If less than 5% of these tests are significant at the 5% level, then unidimensionality is supported [27]. Another approach to examine unidimensionality is to apply a generalization of the Martin-Löf test to the two subsets of items defined previously [28]. A non-significant *p-*value for this test at the 5% level supports the assumption of unidimensionality.

Invariance means that item difficulties remain the same across different groups, such as age or gender. The invariance of items is assessed through an analysis of variance of the residuals where the key group of interest is the main factor. If the inter-person-group variance is statistically significant, the item bias is called Differential Item Functioning [29,30]. When it is present, the probability of an item response cannot be explained totally by the person and item parameters, as it is also influenced by other group properties such as age and gender. Here, each item was checked for Differential Item Functioning across two subgroups: gender (male and female), age (younger or older than 45 years).

Once the three assumptions of local independence, unidimensionality and invariance are met, it is possible to use the Rasch model to further evaluate the scale by investigating overall goodness-of-fit, reliability, fitness of individuals or items, and consistency of items, as introduced following:

#### Overall goodness-of-fit

Most publications dealing with Rasch analysis estimate the overall goodness-of-fit using a chi-square test [22,23,31]. If the data fit the Rasch Model, a summary chi-square interaction statistic should be non-significant. However, recent studies show that chi-square approaches are problematic: these indices are too powerful and the appropriate degree of freedom is often not clear [32,33]. Instead, the Andersen’s likelihood-ratio test [34] shows high power and acceptable type-I error rate in Rasch Model estimation [35]. To perform this test, subjects are split into *g* = 1, …, *G* score-level subgroups in which a conditional likelihood is computed and compared to the total conditional likelihood computed in the complete sample of subjects. The statistic of the test is given by:

$$2\left(\Sigma_{g=1}^{G}logL_{C}^{\left(g\right)}-logL_{C}\right),$$

where $L_{C}^{\left(g\right)}$ is the conditional likelihood of subgroup *g* and *L*_*C*_ is the total conditional likelihood. This statistic has an asymptotic chi-square distribution with degrees of freedom equal to the number of parameters estimated in the score groups minus the number of parameters estimated in the complete data set. A non-significant *p-*value for this test indicates goodness-of-fit for the Rasch model.

Reliability of the CMTNS scale is estimated by the Person Separation Index (PSI) given by the proportion of true variance relative to the true and error variance. In practice it measures the internal consistency and the discrimination power of the scale, i.e. the ability of the scale to discriminate amongst persons with different levels of the trait. It is equivalent to the Cronbach’s alpha [36], but it uses the person estimates in logits instead of the raw scores. A PSI value greater than 0.7 is considered as acceptable.

Item fit can be assessed by several indicators. The residual item fit statistics are expected to approximate a Normal distribution (mean close to 0 with a SD close to 1), which is tested using a chi-square test [21]. A significant chi-square test based *p*-value may indicate misfit. In parallel, a similar analysis could be performed for the test of person fit. Then, fit statistics can be computed and focus on two aspects: infit (means inlier-sensitive fit) and outfit (means outlier-sensitive fit). Infit is more sensitive to the overall pattern and less influenced by outliers and thus infit problems are more of a threat to measurement than outfit ones. Infits and outfits are reported in both mean squares and standardized fit *t*-statistics. The mean squares indicate the amount of distortion of the measurement system whereas the *t*-statistics indicate how likely the item is misfit [37]. Mean-squares greater than 1.3 indicates underfit to the Rasch model, i.e., the data are less predictable than the model expects; mean-squares less than 0.7 indicate overfit to the Rasch model, i.e., the data are more predictable than the model expects. High *t*-statistics (> 2.0) show that the item distorts or degrades the measurement system as underfit while low *t*-statistics (< -2.0) mean data are too predictable or overfit, but not degrading. Underfit and overfit to the model have different implications for measurement. Underfit degrades the quality of the measurement and should prompt reflection on its cause. Overfit might mislead one into concluding that the quality of the measure is better than it really is, and has less practical implication than underfit [38].

#### Consistency of items

A particularly useful output of the Rasch analysis is the person-item map (also sometimes referred to as ‘Wright map’). This map displays the difficulty of the items on the same latent dimension as the impairment of the patients. For each item, a threshold of a category is defined as the location at which the cumulative probability of selecting this category versus all the other options reaches 0.5. In doing so, thresholds should follow the same order as categories. A disorder of categories in an item occurs when the ordinal numbering of categories is not in accord with their fundamental meaning or when individuals have difficulties in consistently discriminating categories. In this case, the disordered categories should be rearranged and Item Characteristic Curves representing the probability of selecting each category for one item can be plotted in order to examine whether this disorder item from under or over-selection of one category.

### Implementation

The Rasch Model has various mathematical variations. Here, we precisely considered the Partial Credit Model [37] allowing different response format for each item, which is the case of the CMTNS. A more detailed introduction to the Partial Credit Model can be found in Wang *et al* [39]. Analyses were performed with *R* (http://cran.r-project.org). The dimensionality, local dependency and invariance analyses were carried out using custom-made *R* functions, while the other Rasch analyses were performed with the *R* package *eRm* [40]. Statistical significance was considered at the 5% level and Bonferroni correction for multiple testing was applied where appropriate.

## Results

We performed a Rasch analysis of the CMTNS using responses from the 277 individuals included in the study. A well targeted sample size of at least 150 individuals is required to reach a 99% confidence that the estimated item difficulty is within +/-0.5 logit of its stable value [41]. Our sample of 277 CMT1A patients was therefore adequate for the analysis. A preliminary quality control based on a significant *p*-value of the person fit chi-square test excluded 15 individuals (5.4%). From there, 262 individuals were included in the Rasch analysis.

### Local independency, unidimensionality and invariance

**Fig 1:**
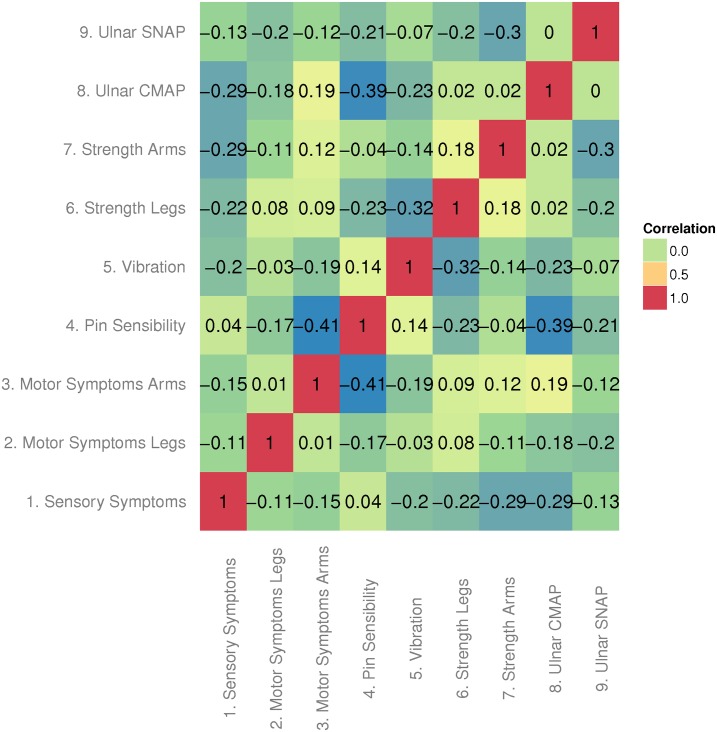
Pairwise correlations between item fit residuals. Pearson correlation coefficients between the residuals of 9 items in the CMTNS. CMAP = Amplitudes of Compound Muscle Action Potentials; SNAP = Amplitudes of Sensory Nerve Action Potentials.

We investigated the compliance of the CMTNS to the main assumptions of the Rasch model. Firstly, local independency was shown by the absence of pairwise correlations between item fit residuals greater than 0.3 (Fig 1). Then, unidimensionality was supported by the fact that only 2 patients of 262 total (much less than 5%) had a significant *p*-value following the PCA approach described in the methods. The *p*-value of the Martin-Löf test was not significant (*p* = 0.919). Finally, the response residuals of different subgroups (gender, age) in each item do not display significant Differential Item Functioning, which means invariance of items. These results led us to conclude that the CMTNS meet the assumptions of the Rasch Model in our cohort of CMT1A patients.

### Overall goodness-of-fit and reliability

A non-significant *p*-value of the Andersen’s likelihood-ratio test (*p* = 0.435) indicates a good overall fit of the CMTNS to the Rasch model. The PSI calculated on our data equals 0.715, pointing to acceptable reliability of the CMTNS, although this value is not particularly high.

### Item fit

**Fig 2:**
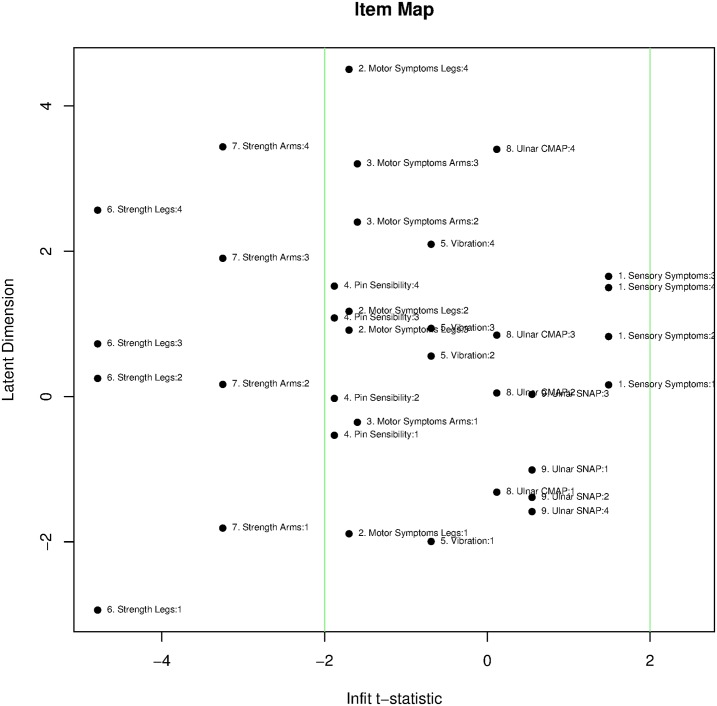
Infit statistics of categories of each item. The *x* axis displays the infit *t*-statistics of each category in 9 items. The *y* axis represents category distributions on the logit scale of latent dimension. Most items fall within the range -2 to 2 (indicated by green lines). CMAP = Amplitudes of Compound Muscle Action Potentials; SNAP = Amplitudes of Sensory Nerve Action Potentials.

On the item level, ‘*Ulnar SNAP*’ is the only item of the CMTNS that has a significant chi-square based *p-*value at the 5% level (*p* = 0.044). However, it is not significant after Bonferroni correction. Residuals of all items have a distribution with means close to 0 and SD close to 1 (see Table 1). None of the infit *t* statistics are superior to 2 (Fig 2), which is to say no item distorts the measurement. Both infit and outfit of the ‘*Strength Legs*’ and ‘*Strength Arms*’ items are inferior to -2, and the fit mean squares of ‘*Strength Legs*’ was slightly lower than 0.7, which points to responses to the two items as being too predictable, possibly leading to overfit.

**Table 1:** Item statistics. MSQ = Mean-square.

Item names	Location	Residual mean	Residual SD	*p*-value	Outfit MSQ	Infit MSQ	Outfit *t*-stat	Infit *t*-stat
**1. Sensory Symptoms**	0.806	0.001	1.049	0.128	1.096	1.138	0.964	1.491
**2. Motor Symptoms Legs**	0.942	-0.031	0.924	0.958	0.851	0.862	-1.675	-1.699
**3. Motor Symptoms Arms**	1.54	-0.016	0.931	0.941	0.864	0.861	-1.616	-1.597
**4. Pin Sensibility**	0.429	0.001	0.943	0.902	0.885	0.856	-1.431	-1.88
**5. Vibration**	0.353	-0.014	0.966	0.775	0.929	0.941	-0.793	-0.69
**6. Strength Legs**	0.105	-0.034	0.81	1	0.654	0.661	-4.689	-4.789
**7. Strength Arms**	0.755	-0.008	0.864	0.999	0.744	0.745	-3.28	-3.251
**8. Ulnar CMAP**	0.687	0.010	1.031	0.23	1.059	1.007	0.76	0.116
**9. Ulnar SNAP**	-0.788	0.018	1.074	0.044	1.15	1.054	0.86	0.55

### Consistency of items

**Fig 3:**
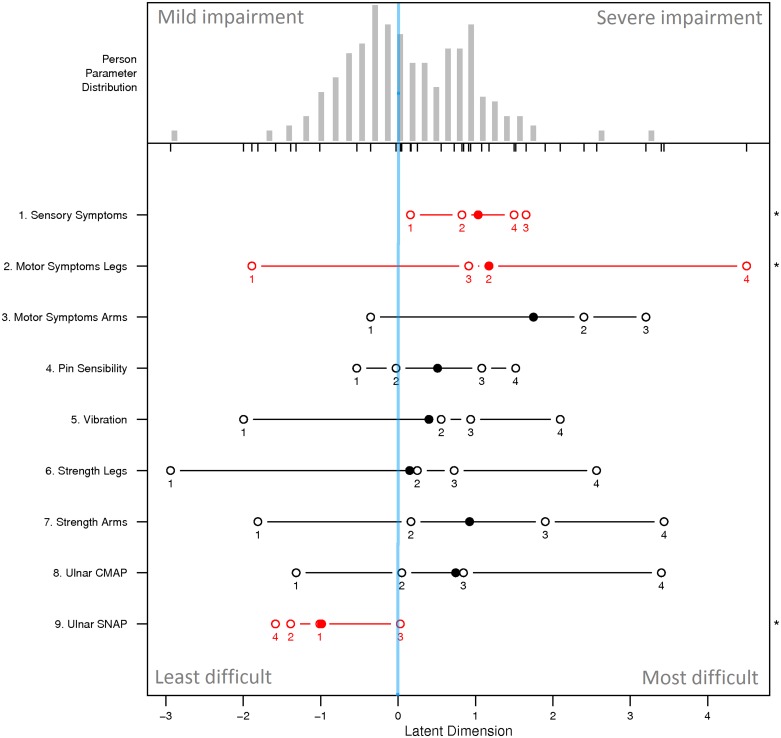
Person-Item map. The person-item map displays the location of person abilities and item difficulties respectively along the same latent dimension. The person parameter is located on the scale from left (mild impairment) to right (severe impairment). Locations of item difficulties are displayed with solid circles and thresholds of adjacent category locations with open circles. Items with disordered thresholds are marked in red and asterisks. The blue line indicates the zero level of the latent trait.

**Fig 4:**
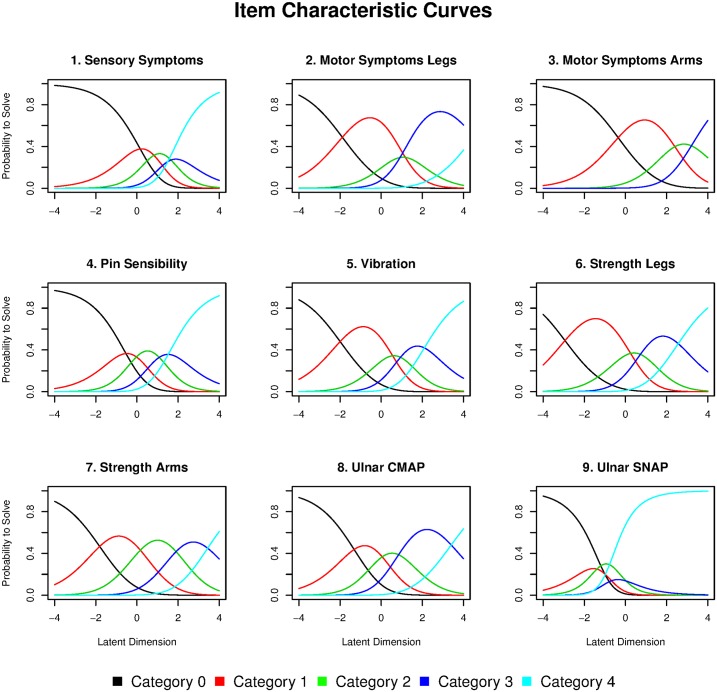
Item Characteristic Curves. Curves represent the probability of selecting a category in an item.

The person-item map (Fig 3) displays the location of person abilities and item difficulties respectively along the same latent dimension. Although the category thresholds of most items cover mild-to-severe range of disability well, item difficulty locations clump at the range of patients with higher person parameters (right side of the latent dimension), which means that they have more probability to differentiate patients with higher level of disease severity. For instance, ‘*Motor Symptom Arm*’ shows the highest item difficulty meaning that mild-to-moderate patients are more likely to answer ‘0’ (i.e. no disability in arms) for this item. Three items have disordered categories (‘*Sensory Symptoms*’, ‘*Motor Symptoms Legs*’ and ‘*Ulnar SNAP*’) indicated in red on the person-item map (Fig 3). To further investigate these disordered items, we examined the Item Characteristic Curves (Fig 4). Category 2 in ‘*Motor Symptoms Legs*’ (*i*.*e*. ankle-foot orthosis on at least one leg or ankle support) was under-selected, which causes the observed disorder. ‘*Sensory Symptoms*’ and ‘*Ulnar SNAP*’ have the categories 0 and 4 evidently over-selected compared to other categories, which means that they are not adapted to discriminate CMT1A patients well.

### Proposed modification of the CMTNS

**Fig 5:**
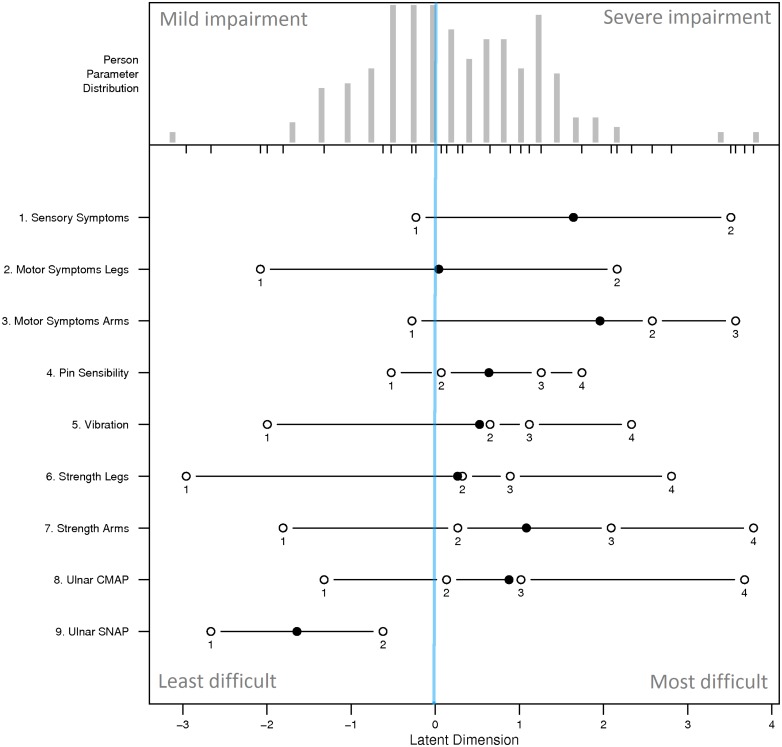
Person-Item map of the CMTNS after modification.

In attempts to improve item fit to the model, a common strategy is to collapse adjacent categories when they have disordered thresholds. Given our results, we collapsed Categories 2, 3 and 4 into one category in ‘*Sensory Symptoms*’ and ‘*Ulnar SNAP*’ and Categories 2, 3 in ‘*Motor Symptoms Legs*’. The person-item map of the modified data shows that all items are now well-ordered (Fig 5). However, after these modifications, the PSI of the CMTNS does not improve (= 0.713 now), and the infit *t*-statistics of ‘*Sensory Symptoms*’ increased from 1.49 to 1.88. Although item categories are well ordered after our modifications, this modification does not enhance the overall fitness of the CMTNS to the Rasch Model.

## Discussion

The CMTNS was developed by Shy *et al*. [15] as the first composite clinical scale dedicated to quantifying impairment and measuring progression in CMT patients. Although the validity of CMTNS to assess severity has never been questioned, its sensitivity to change and its ability measure a response to treatment are still debated. Subsequent versions of the CMTNS have been proposed, such as the CMTNS-v2 by Murphy *et al*. [42] to attempt to reduce floor and ceiling effects, the CMTNS-Mod by Mannil *et al*. [19] by adding three functional measures while removing three of the initial items, and finally a ‘weighted’ alternative of the CMTNS-v2 by Sadjadi *et al*. [18] resulting from a Rasch analysis. None of these modified versions have been evaluated in natural history or therapeutic trials.

In order to further investigate some key properties of the original CMTNS and to identify possible directions of improvement, we performed a validation of this scale based on a Rasch analysis in a cohort of 277 mild-to-severe CMT1A French patients, made possible by the integration of 3 studies including 2 published clinical trials [9,14]. Our first result is that overall and in the context of the Rasch analysis, the CMTNS appears as a valid measurement for CMT1A: the three main assumptions of the Rasch model (local independency, unidimensionality and invariance) were met, the scale showed good overall fit to the Rasch model and an acceptable measure of reliability. When analyzed individually, only two items (‘*Strength Legs*’ and ‘*Strength Arms*’) showed an overfit to the model (infit and outfit *t*-statistics < -2), which has little major implication for the quality of the measurement. In the Rasch analysis of Sadjadi *et al*. [18] of the CMTNS-v2, all of the items showed good fit supporting the idea that they belong in the scale and contribute to the overall score of impairment. Although the CMTNS-v2 presents some modifications to the CMTNS in terms of categories or instruments of measure, they are very similar and can be discussed together here.

As a limitation, the person-item map suggests that the items of the CMTNS are more suitable for assessing moderate to severe forms of the disease, with the exception of ‘*Ulnar SNAP’*. Sadjadi *et al*. [18] arrived at the same conclusion, though in the CMTNS-v2, SNAP is measured on the radial nerve instead of the ulnar nerve, underlining the consistency of this result. This finding is also supported by a comparison study of the CMTNS-v2 and a pediatric version of the CMTNS (called CMTPedS and proposed in Burns *et al*. [31]) where the authors observed a lack of sensitivity of the CMTNS-v2 for assessing mild patients [43].

Finally, we found that 3 items (‘*Sensory Symptoms*’, ‘*Motor Symptoms Legs*’ and ‘*Ulnar SNAP*’) had disordered categories, meaning that they are not adapted to discriminate CMT1A patients well. Category 2 in ‘*Motor Symptoms Legs*’ (i.e. ankle-foot orthosis on at least one leg or ankle support) was under-selected while extreme categories (0 and 4) in items ‘*Sensory Symptoms*’ and ‘*Ulnar SNAP*’ were evidently over-selected. A slight modification of the CMTNS by collapsing the disordered categories corrected this problem but did not improve the overall fit to the Rasch model.

In conclusion, the choice of clinical endpoints for assessing disease severity, progression and response to treatment remains an active topic in the field of chronic neuromuscular diseases. In this context, the CMTNS (first and second versions) is the only clinical scale specific to CMT and as such, has been widely applied in natural history and therapeutic trials. In light of our results and a review of the literature, it is clear that the choice of applying the CMTNS as a measure of severity, disease progression or therapeutic efficacy in clinical practice is a choice to be made after careful consideration. Our current position is that, by integrating different components of the disease, the CMTNS remains an appropriate measure of impairment, particularly useful for classifying patients into mild, moderate and severe. Finally, further refinement of the CMTNS and/or its modified versions is certainly worth consideration in order to overcome the limitations identified here and move towards an optimal scale.

## References

[1] Kochański A. Molecular genetics studies in Polish Charcot-Marie-Tooth families. Folia Neuropathol. 2005;43: 65–73. 16012907

[2] Patzkó Á, Shy ME. Update on Charcot-Marie-Tooth disease. Curr Neurol Neurosci Rep. 2011;11: 78–88. 10.1007/s11910-010-0158-7 21080241 PMC3685483

[3] Raeymaekers P, Timmerman V, Nelis E, De Jonghe P, Hoogendijk JE, Baas F, et al Duplication in chromosome 17p11.2 in Charcot-Marie-Tooth neuropathy type 1a (CMT 1a). The HMSN Collaborative Research Group. Neuromuscul Disord. 1991;1: 93–97. 1822787 10.1016/0960-8966(91)90055-w

[4] Lupski JR, Wise CA, Kuwano A, Pentao L, Parke JT, Glaze DG, et al Gene dosage is a mechanism for Charcot-Marie-Tooth disease type 1A. Nat Genet. 1992;1: 29–33. 10.1038/ng0492-29 1301995

[5] Foley C, Schofield I, Eglon G, Bailey G, Chinnery PF, Horvath R. Charcot-Marie-Tooth disease in Northern England. J Neurol Neurosurg Psychiatry. 2011;83: 572–573. 10.1136/jnnp-2011-300285 21984771

[6] Verhamme C, de Haan R, Vermeulen M, Baas F, de Visser M, van Schaik I. Oral high dose ascorbic acid treatment for one year in young CMT1A patients: a randomised, double-blind, placebo-controlled phase II trial. {BMC} Med. 2009;7: 70 10.1186/1741-7015-7-70 19909499 PMC2784478

[7] Burns J, Ouvrier RA, Yiu EM, Joseph PD, Kornberg AJ, Fahey MC, et al Ascorbic acid for Charcot-Marie-Tooth disease type 1A in children: a randomised, double-blind, placebo-controlled, safety and efficacy trial. Lancet Neurol. 2009;8: 537–544. 10.1016/S1474-4422(09)70108-5 19427269

[8] Toth C. Poor tolerability of high dose ascorbic acid in a population of genetically confirmed adult Charcot-Marie-Tooth 1A patients. Acta Neurologica Scandinavica. 2009 pp. 134–138. 10.1111/j.1600-0404.2008.01134.x 19154534

[9] Micallef J, Attarian S, Dubourg O, Gonnaud P-M, Hogrel J-Y, Stojkovic T, et al Effect of ascorbic acid in patients with Charcot-Marie-Tooth disease type 1A: a multicentre, randomised, double-blind, placebo-controlled trial. Lancet Neurol. 2009;8: 1103–1110. 10.1016/S1474-4422(09)70260-1 19818690

[10] Pareyson D, Reilly MM, Schenone A, Fabrizi GM, Cavallaro T, Santoro L, et al Ascorbic acid in Charcot-Marie-Tooth disease type 1A (CMT-TRIAAL and CMT-TRAUK): a double-blind randomised trial. Lancet Neurol. 2011;10: 320–8. 10.1016/S1474-4422(11)70025-4 21393063 PMC3154498

[11] Lewis RA, McDermott MP, Herrmann DN, Hoke A, Clawson LL, Siskind C, et al High-dosage ascorbic acid treatment in Charcot-Marie-Tooth disease type 1A: results of a randomized, double-masked, controlled trial. JAMA Neurol. 2013;70: 981–7. 10.1001/jamaneurol.2013.3178 23797954 PMC3752369

[12] Mandel J, Bertrand V, Lehert P, Attarian S, Magy L, Micallef J, et al A meta-analysis of randomized double-blind clinical trials in CMT1A to assess the change from baseline in CMTNS and ONLS scales after one year of treatment. Orphanet J Rare Dis. 2015;10: 74 10.1186/s13023-015-0293-y 26070802 PMC4482281

[13] Gess B, Baets J, De Jonghe P, Reilly MM, Pareyson D, Young P. Ascorbic acid for the treatment of Charcot-Marie-Tooth disease. Cochrane database Syst Rev. 2015;12: CD011952. 10.1002/14651858.CD011952 PMC6823270 26662471

[14] Attarian S, Vallat J-M, Magy L, Funalot B, Gonnaud P-M, Lacour A, et al An exploratory randomised double-blind and placebo-controlled phase 2 study of a combination of baclofen, naltrexone and sorbitol (PXT3003) in patients with Charcot-Marie-Tooth disease type 1A. Orphanet J Rare Dis. 2014;9: 199 10.1186/s13023-014-0199-0 25519680 PMC4311411

[15] Shy ME, Blake J, Krajewski K, Fuerst DR, Laura M, Hahn AF, et al Reliability and validity of the CMT neuropathy score as a measure of disability. Neurology. 2005;64: 1209–14. 10.1212/01.WNL.0000156517.00615.A3 15824348

[16] Reilly MM, Shy ME, Muntoni F, Pareyson D. 168th ENMC International Workshop: outcome measures and clinical trials in Charcot-Marie-Tooth disease (CMT). Neuromuscul Disord. 2010;20: 839–46. 10.1016/j.nmd.2010.08.001 20850975

[17] Murphy SM, Herrmann DN, McDermott MP, Scherer SS, Shy ME, Reilly MM, et al Reliability of the CMT neuropathy score (second version) in Charcot-Marie-Tooth disease. J Peripher Nerv Syst. 2011;16: 191–198. 10.1111/j.1529-8027.2011.00350.x 22003934 PMC3754828

[18] Sadjadi R, Reilly MM, Shy ME, Pareyson D, Laura M, Murphy S, et al Psychometrics evaluation of Charcot-Marie-Tooth Neuropathy Score (CMTNSv2) second version, using Rasch analysis. J Peripher Nerv Syst. 2014;19: 192–6. 10.1111/jns.12084 25400013 PMC4303498

[19] Mannil M, Solari A, Leha A, Pelayo-Negro AL, Berciano J, Schlotter-Weigel B, et al Selected items from the Charcot-Marie-Tooth (CMT) Neuropathy Score and secondary clinical outcome measures serve as sensitive clinical markers of disease severity in CMT1A patients. Neuromuscul Disord. 2014;24: 1003–17. 10.1016/j.nmd.2014.06.431 25085517

[20] Shy ME, Chen L, Swan ER, Taube R, Krajewski KM, Herrmann D, et al Neuropathy progression in Charcot-Marie-Tooth disease type 1A. Neurology. 2008;70: 378–383. 10.1212/01.wnl.0000297553.36441.ce 18227419

[21] Kersten P, White PJ, Tennant A. Is the pain visual analogue scale linear and responsive to change? An exploration using rasch analysis. PLoS One. 2014;9. 10.1371/journal.pone.0099485 PMC4055724 24921952

[22] Tennant A, Conaghan PG. The Rasch measurement model in rheumatology: What is it and why use it? When should it be applied, and what should one look for in a Rasch paper? Arthritis Care Res. 2007;57: 1358–1362. 10.1002/art.23108 18050173

[23] Zucca AC, Lambert SD, Boyes AW, Pallant J. Rasch analysis of the Mini-Mental Adjustment to Cancer Scale (mini-MAC) among a heterogeneous sample of long-term cancer survivors: A crosssectional study. Health and Quality of Life Outcomes. 2012 p. 55 10.1186/1477-7525-10-55 22607052 PMC3487859

[24] van Nes SI, Vanhoutte EK, van Doorn P a, Hermans M, Bakkers M, Kuitwaard K, et al Rasch-built Overall Disability Scale (R-ODS) for immune-mediated peripheral neuropathies. Neurology. 2011;76: 337–45. 10.1212/WNL.0b013e318208824b 21263135

[25] Fischer GH. Rasch Models [Internet]. Fischer GH, Molenaar IW, editors. New York, NY: Springer New York; 1995.

[26] Andrich D. Sufficiency and conditional estimation of person parameters in the Polytomous Rasch Model. Psychometrika. 2010;75: 292–308.

[27] Smith E V. Detecting and evaluating the impact of multidimensionality using item fit statistics and principal component analysis of residuals. J Appl Meas. 2002;3: 205–231. 12011501

[28] Christensen KB, Bjorner JB, Kreiner S, Petersen JH. Testing unidimensionality in polytomous Rasch models. Psychometrika. 2002 pp. 563–574.

[29] Hanson BA. Uniform DIF and DIF Defined by Differences in Item Response Functions. Journal of Educational and Behavioral Statistics. 1998 pp. 244–253.

[30] Kim SH, Cohen AS, Alagoz C, Kim S. DIF detection and effect size measures for polytomously scored items. J Educ Meas. 2007;44: 93–116.

[31] Burns J, Ouvrier R, Estilow T, Shy R, Laurá M, Pallant JF, et al Validation of the Charcot-Marie-Tooth disease pediatric scale as an outcome measure of disability. Ann Neurol. 2012;71: 642–52. 10.1002/ana.23572 22522479 PMC3335189

[32] Orlando M, Thissen D. Likelihood-Based Item-Fit Indices for Dichotomous Item Response Theory Models. Applied Psychological Measurement. 2000 pp. 50–64.

[33] Orlando M, Thissen D. Further Investigation of the Performance of S—X2: An Item Fit Index for Use With Dichotomous Item Response Theory Models. Applied Psychological Measurement. 2003 pp. 289–298.

[34] Andersen EB. A goodness of fit test for the rasch model. Psychometrika. 1973;38: 123–140.

[35] Suárez-Falcón JC, Glas CAW. Evaluation of global testing procedures for item fit to the Rasch model. Br J Math Stat Psychol. 2003;56: 127–143. 10.1348/000711003321645395 12803827

[36] Cronbach LJ. Coefficient alpha and the internal structure of tests. Psychometrika. 1951;16: 297–334.

[37] Masters GN. A Rasch model for partial credit scoring. Psychometrika. Springer; 1982;47: 149–174. http://www.springerlink.com/index/5000VV4281622815.pdf

[38] Green K, Frantom C. Survey development and validation with the Rasch model. Int Conf Quest Dev Eval Test. 2002; 1–30.

[39] Wang W, Mandel J, Bouaziz J, Commenges D, Nabirotchkine S, Chumakov I, et al A Multi-Marker Genetic Association Test Based on the Rasch Model Applied to Alzheimer’s Disease. PLoS One. Public Library of Science; 2015;10: e0138223. 10.1371/journal.pone.0138223 PMC4574966 26379234

[40] Mair P, Hatzinger R. Extended Rasch Modeling: The R Package eRm. 2007;

[41] Linacre JM. WINSTEPS Rasch measurement computer program. Chicago Winsteps com. 2006;

[42] Murphy SM, Herrmann DN, McDermott MP, Scherer SS, Shy ME, Reilly MM, et al Reliability of the CMT neuropathy score (second version) in Charcot-Marie-Tooth disease. J Peripher Nerv Syst. 2011;16: 191–8. 10.1111/j.1529-8027.2011.00350.x 22003934 PMC3754828

[43] Burns J. Transitioning outcome measures: relationship between the CMTPedS and CMTNSv2 in children, adolescents, and young adults with Charcot-Marie-Tooth disease. J Peripher Nerv Syst. 2013;18, 2. 10.1111/jns5.12024 PMC3714225 23781965

